# Vertical structure in chlorophyll profiles: influence on primary production in the Arctic Ocean

**DOI:** 10.1098/rsta.2019.0351

**Published:** 2020-08-31

**Authors:** Heather A. Bouman, Thomas Jackson, Shubha Sathyendranath, Trevor Platt

**Affiliations:** 1 Department of Earth Sciences, University of Oxford, South Parks Road, Oxford OX1 3AN, UK; 2 Plymouth Marine Laboratory, Remote Sensing Unit, Plymouth, UK

**Keywords:** Arctic Ocean, primary production, subsurface chlorophyll maximum

## Abstract

Subsurface chlorophyll maximum (SCM) layers are prevalent throughout the Arctic Ocean under stratified conditions and are observed both in the wake of retreating sea ice and in thermally stratified waters. The importance of these layers on the overall productivity of Arctic pelagic ecosystems has been a source of debate. In this study, we consider the three principal factors that govern productivity within SCMs: the shape of the chlorophyll profile, the photophysiological characteristics of phytoplankton and the availability of light in the layer. Using the information on the biological and optical parameters describing the vertical structure of chlorophyll, phytoplankton absorption and photosynthesis–irradiance response curves, a spectrally resolved model of primary production is used to identify the set of conditions under which SCMs are important contributors to water-column productivity. Sensitivity analysis revealed systematic errors in the estimation of primary production when the vertical distribution of chlorophyll was not taken into account, with estimates of water-column production using a non-uniform profile being up to 97% higher than those computed using a uniform one. The relative errors were shown to be functions of the parameters describing the shape of the biomass profile and the light available at the SCM to support photosynthesis. Given that SCM productivity is believed to be largely supported by new nutrients, it is likely that the relative contribution of SCMs to new production would be significantly higher than that to gross primary production. We discuss the biogeochemical and ecological implications of these findings and the potential role of new ocean sensors and autonomous underwater vehicles in furthering the study of SCMs in such highly heterogeneous and remote marine ecosystems.

This article is part of the theme issue ‘The changing Arctic Ocean: consequences for biological communities, biogeochemical processes and ecosystem functioning'.

## 1. Introduction

The influence of melting sea ice on the density structure in the upper ocean strongly impacts the availability of the two major factors influencing phytoplankton growth: the access to light and the supply of nutrients. The formation of a fresh and relatively thin surface mixed layer caused by ice melt leads to the formation of shallow (less than 20 m) and at times intense (greater than 1 mg chlorophyll m^−3^) subsurface chlorophyll maximum (SCM) layers. Here and elsewhere in the paper ‘chlorophyll’ refers to chlorophyll-a (and the sum of chlorophyll-a and its derivatives when such information is available from HPLC measurements). The overall importance of these chlorophyll layers to annual water-column production in Arctic waters has been debated in the literature [1–4]. It has been argued that when compared with highly productive Arctic Ocean spring blooms, the productivity within SCMs during the post-bloom period is relatively low [4] and that errors introduced by ignoring the presence of SCMs are small when primary production is integrated over large spatial and temporal scales [2,5].

Our ability to assess the biogeochemical and ecological importance of these layers in Arctic seas is frustrated by a number of factors. First, the complex vertical structure of SCMs in Arctic seas is difficult to predict using surface ocean observables such as chlorophyll concentration, although regional [6] and pan-Arctic [4] algorithms have been proposed based on *in situ* datasets. Second, the persistence of these layers within Arctic marine ecosystems is poorly known. This is largely a consequence of the inaccessibility of Arctic waters to routine oceanographic sampling by ship and our inability to observe these layers from space. Consequently, our current knowledge of the seasonal and geographical distribution of SCMs is largely based on *in situ* measurements spanning decades [7], and these datasets lack consistency in sampling approach and have uneven spatial (both in the vertical and in the horizontal) and temporal (seasonal) coverage. Lastly, as a direct result of the sparsity of chlorophyll data in the Arctic Ocean, our estimates of the contribution of SCMs to water-column primary production often involve using compilations of *in situ* chlorophyll measurements and partitioning them according to ecological provinces [5]; or the surface chlorophyll concentration and bloom phase [4]; or region and degree of ice cover [3]; or geographical sector [2]. By using averaged depth-binned profiles to represent regional and seasonal variability in the vertical structure of chlorophyll, information on the diversity of SCM shape is eroded and the resulting averaged profiles are smoothed and flattened. Yet sensitivity analysis has demonstrated that the impact of chlorophyll layers on estimates of water-column primary production depends on both the sharpness and the location of the chlorophyll peak in relation to the photic depth [8].

In the Arctic Ocean, the impact of surface-water freshening from ice melt results in intense vertical stratification and a shallow pycnocline, setting up strong opposing gradients of light and nutrient availability. The SCM under these conditions is often in close proximity to the nitracline [9] and can be situated well above the photic depth (*z_p_*) [1], whereas the deep chlorophyll maximum, which is a ubiquitous feature in lower-latitude open-ocean waters and is sometimes referred to as the ‘typical tropical’ structure, tends to coincide with *z_p_* [10,11]. A direct consequence of an illuminated SCM is that its fractional contribution to integrated water-column production can be significant depending on the photosynthetic characteristics of the resident microalgae [1,8].

For the polar biome of the North Atlantic, Sathyendranath and co-workers [5] reported that the effect of the SCM was less than 10% in the Arctic, and less than 4% in the SubArctic and Boreal Polar provinces, at the annual scale. However, they argued that this contribution, though modest, would always introduce a negative bias in computed primary production if ignored (inclusion of a subsurface peak in the calculation typically increases the magnitude of the computed water-column production), thereby affecting the accuracy, rather than the precision of the computed value. Furthermore, they pointed out that production in the subsurface peak is likely associated with the supply of new nutrients from below the mixed layer, and hence important when assessing new production, which is more relevant in climate studies. Twenty-five years on, the information on the vertical structure in chlorophyll concentration has certainly increased substantially, and it is worth reassessing the contribution of the subsurface chlorophyll peaks in the Arctic to the water-column primary production.

To examine the overall contribution of SCM layers to integrated water-column primary production requires not only accounting for the vertical variation in chlorophyll concentration, but also the photophysiological properties of the cells and the availability of light [8]. In other words, whether the rate of carbon fixation is close to the optimal rate (governed by the plateau of the PE response curve $P_{m}^{B}$) or in the linear response (governed by the initial slope *α*^*B*^) is dictated by the light intensity at a particular depth and time. The parameters used to mathematically describe the photosynthesis–irradiance (PE) response curves [12] not only provide critical information for light-driven primary production models but also serve as important ecophysiological indicators of the ocean's microflora [13]. High-latitude polar seas are typically characterized by lower chlorophyll-specific rates of carbon fixation at saturating light intensities represented by the assimilation number $P_{m}^{B}$ [14,15]. These low rates of chlorophyll-normalized maximum photosynthesis have been attributed in part to the metabolic costs of living in persistently cold ecosystems [16].

Satellite-based calculations of pan-Arctic marine primary production use either a single value of $P_{m}^{B}$ or *α*^*B*^ or allow them to vary seasonally within oceanic provinces [5,17] or assign them dynamically using other satellite observables [18]. Although changes in the photophysiological properties of Arctic phytoplankton have been widely documented in historical datasets [19–21], the lack of ancillary data has restricted the development of a mechanistic approach to parameter assignment. Even though new datasets are being collected to help relate the variability of PE parameters to phytoplankton community structure, light history and nutrient availability [15,22,23], our understanding of the taxonomically and environmentally driven variation in the photosynthetic utilization of light by phytoplankton is still limited to individual oceanographic campaigns. Thus, in order to ascertain the role of SCM in the Arctic Ocean carbon cycle, we need to also examine how sensitive primary production models are to the assignment of parameters used to describe the photosynthetic performance of marine phytoplankton.

In this study, we use datasets from cruises in the Atlantic sector of the Arctic Ocean to examine the natural variability in the key parameters used to describe the chlorophyll profile and phytoplankton photophysiology. Using these data alongside information on the optical properties of Arctic marine phytoplankton, a vertically and spectrally resolved model of water-column primary production is used to determine how relevant SCMs are to our estimates of integrated primary production and under what circumstances are they important. We conclude by making the case for further studies of biogeochemistry and ecology of SCMs in a changing Arctic Ocean and the role new autonomous sensors may play in facilitating this undertaking.

## 2. Material and Methods

In general, there are three things that must be known to calculate the amount of carbon fixed by marine photoautotrophs. First, how much photosynthetically available light is present; second, how much plant pigment is present to intercept this light; and third, the efficiency with which light absorbed by these pigments is converted into the photosynthetic substrate (carbon). Since phytoplankton pigments and non-algal dissolved and particulate absorptive components are strong attenuators of light, estimation of the underwater light field requires knowledge of both the magnitude and spectral dependence of absorption by these materials. In this study, we used measurements of phytoplankton pigment concentrations, light absorption coefficients and PE parameters collected from a series of cruises in the Atlantic sector of the Arctic Ocean (Canadian Archipelago, Labrador, Greenland/Norwegian and Barents Seas).

### (a) Chlorophyll profile parameters

Information on both the shape and magnitude of the SCM is critical to predict both the underwater light field and water-column primary production. Since the information on chlorophyll concentration obtained by satellite is heavily weighted toward the sea surface [3], we must rely on *in situ* profiles of pigment concentration to parameterize the vertical structure of SCMs. The shape of the phytoplankton biomass profiles can be expressed as a standard function of depth using the following equation [8]:

2.1 $$B(z)=B_{0}+\frac{h}{\sigma \sqrt{2\pi }}\exp\left[-\frac{{\left(z-z_{m}\right)}^{2}}{2\sigma^{2}}\right],$$

where B(z) is chlorophyll biomass as a function of depth *z*; *z_m_* is the centre of the Gaussian peak (corresponding to the depth of the maximum chlorophyll concentration); and *σ* and *h* are related to the width and area of the peak, respectively. The four-parameter Gaussian function [8] was fitted to chlorophyll measurements taken from both discrete bottle data or *in vivo* fluorescence profiles calibrated against *in vitro* chlorophyll extracts. In the case of the bottle data, only chlorophyll measurements from stations where greater than six discrete depths which captured the vertical shape of the profile were used. In the case of the calibrated fluorescence profiles, we recognize that photochemical quenching may have been present at the sea surface. We derived a correction factor that accounts for this potential underestimation in chlorophyll concentration that was observed within the top 20 m in some of the fluorescence profiles by fitting a linear equation to the log-transformed chlorophyll and *in vivo* fluorescence data. The linear equation fit provides the correction parameter (Δ*B*), which we used to adjust the estimates of chlorophyll concentration based on the fluorescence trace close to the sea surface.

2.2 $$\Delta B=xB{\left(z\right)}^{y}-B(z).$$

Since the degree of quenching is strongly related to the irradiance field, we multiplied Δ*B* by an exponential decay function with a coefficient of 0.05:

2.3 B(z)=B(z)+ΔBexp⁡(−0.05z).

### (b) Light absorption spectra

The *in vivo* absorption spectrum of marine phytoplankton was determined using the filter-pad technique [24]. Seawater samples were filtered through 25 mm GF/F (Whatman) filters using a vacuum filtration pump under low pressure. The optical density of total particulates retained on the filter pad $O_{t}(\lambda )$ was measured using a dual-beam spectrophotometer (Shimadzu Corp., UV-2101, Kyoto) equipped with an integrating sphere. Pigments were then extracted by passing 20 ml of hot methanol through the filters [24]. The filters were then rinsed with filtered seawater and re-scanned to obtain the optical density of the detrital component $\left(O_{d}(\lambda )\right)$. Optical densities were then converted to absorption coefficients [25] and the absorption by phytoplankton pigments $a_{p}(\lambda )$ was determined by subtracting the absorption by detrital material from the total absorption by particulates.

In addition to the light absorption by algal and non-algal particulates, coloured dissolved organic matter (CDOM) can also have a strong impact on the spectral quality and intensity of the underwater light field. The shape of the absorption by CDOM (also called yellow substances, denoted by the subscript *y*) is characterized in most bio-optical models as a decreasing exponential function as follows:

2.4 $$a_{y}(\lambda )=a_{y}(440)\exp(-x(\lambda -440)),$$

where *x* is the coefficient that describes how rapid the exponential decreases with wavelength and *a_y_*(440) is the absorption coefficient of yellow substances at 440 nm. For our standard model runs, we applied a spectral slope value of 0.014 nm^−1^ [26] and the magnitude of CDOM absorption was scaled by a coefficient of proportionality between absorption by CDOM at 440 nm (*a_y_*(440)) and the sum of the coefficients of phytoplankton and pure water at 440 nm $\left(a_{\text{ph}}(440)+a_{w}(440)\right)$. For the standard model run, $a_{y}(440)/(a_{\text{ph}}(440)+a_{w}(440))$ was assigned a value of 0.30.

### (c) PE response parameters

Along with an accurate description of the underwater light field, parameters describing the photosynthetic response of marine phytoplankton to available light must be known before the rate of primary production can be estimated. The PE response curve can be described using two parameters: $P_{m}^{B}$, the assimilation number and *α*^*B*^, the initial slope, where the superscript *B* indicates normalization to phytoplankton biomass [12]. To obtain PE response curves, 14–30 light bottles containing 60–100 ml of seawater were spiked with between 10 and 20 μCi of sodium ^14^C-bicarbonate. To maintain the samples at *in situ* temperatures, a temperature-controlled circulating water bath was used. Bottles were then placed in a light gradient ranging from approximately 8–2700 µmol quanta m^−2^ s^−1^ and incubated over 2–3 h. At the end of the incubations, samples were filtered onto GF/F filters. The filters were thoroughly rinsed with filtered seawater and fumed over a bath of concentrated HCl to remove any inorganic ^14^C remaining on the filter. Samples were then counted in a liquid scintillation counter. Primary production per hour, normalized to fluorometrically determined chlorophyll concentrations, was estimated from the scintillation counts. These data were then fitted to an equation, which accounts for photoinhibition to obtain the initial slope (*α*^*B*^) and the photosynthetic rate at saturating irradiance ($P_{m}^{B}$) [27]. Values of *α*^*B*^ were corrected for the emission spectrum of the incubator lamp as described in [28]. The PE parameters dataset (shown in the electronic supplementary material) is a subset of two datasets: a global dataset [14], which is publically available (https://doi.org/10.1594/PANGAEA.874087) and one of the Labrador Sea [29], which is publically available at https://doi.org/10.1594/PANGAEA.871872. Note that only data collected at latitudes greater than 60°N were used in this study.

In the spectral model of marine primary production, the shape of the absorption spectrum was used as a proxy for the action spectrum $\alpha^{B}(\lambda )$ [30]. Pigments that are not photosynthetically active may be present and could lead to a mismatch between the shape of the absorption and action spectra. However, in the Arctic Ocean such pigments are found within surface assemblages, and thus their impact on primary productivity in the light-limited portion of the water column would be negligible [31].

Since both irradiance and the light-limited photosynthetic rate demonstrate strong wavelength dependence, we have represented the spectral characteristics of both of these quantities into our computation of primary production. In PE equations, *α*^*B*^ and *E*(*z*) occur together as a product ($\Pi^{B}$(z)):

2.5 $$\Pi^{B}(z)=\alpha^{B}E(z).$$

Given *α*^*B*^ and *E*(*z*) are both spectrally dependent, they are expressed as functions of wavelength [32]:

2.6 $$\Pi_{\lambda }^{B}=\int_{400}^{700}\alpha^{B}(\lambda )E(z,\lambda )d\lambda .$$

The chlorophyll-normalized photosynthetic rate can, therefore, be defined as follows [33]:

2.7 $$P^{B}(z)=\frac{\Pi_{\lambda }^{B}}{\sqrt{1+{\left(\Pi_{\lambda }^{B}/P_{m}^{B}\right)}^{2}}}.$$

The model also incorporates the angular dependence of the underwater light field as described in [32]. A clear-sky model [34] was used to estimate spectral irradiance incident at the sea surface under cloud-free conditions. To examine the impact of cloud cover on productivity we reduced the magnitude of irradiance based on the percentage daily cloud cover [35], which we varied from 0 (clear-sky) to 100%, and adjusted the diffuse part of irradiance with respect to the direct component accordingly. Rates of primary production were integrated down to the photic depth *z_p_*, which we defined as that depth at which modelled light integrated over the visible domain was reduced to 1% of the integrated surface irradiance.

To examine the impact of the vertical distribution of chlorophyll on estimates of water-column primary production we adopt an approach used in previous studies [3,5,8], where *in situ* chlorophyll profile data are used to capture the natural variability in shape and magnitude of *B*(*z*) and then compute primary production by both using the *in situ* profile shapes and also holding the surface chlorophyll concentration constant over depth. Whereas Hill and co-workers [3] used an average PE response curve based on information on long-term (12–24 h) *in situ* incubations and *in situ* light levels expressed as a percentage of the surface irradiance, our computations use short-term (2–3 h) PE parameters generated under experimentally controlled light conditions and a radiative transfer model that incorporates the angular and spectral distribution of the underwater light field. Another important distinction between this study and that of Hill and co-workers [3] is that individual profiles are used rather than averaged depth-binned profiles.

## 3. Results and discussion

**Figure 1.**
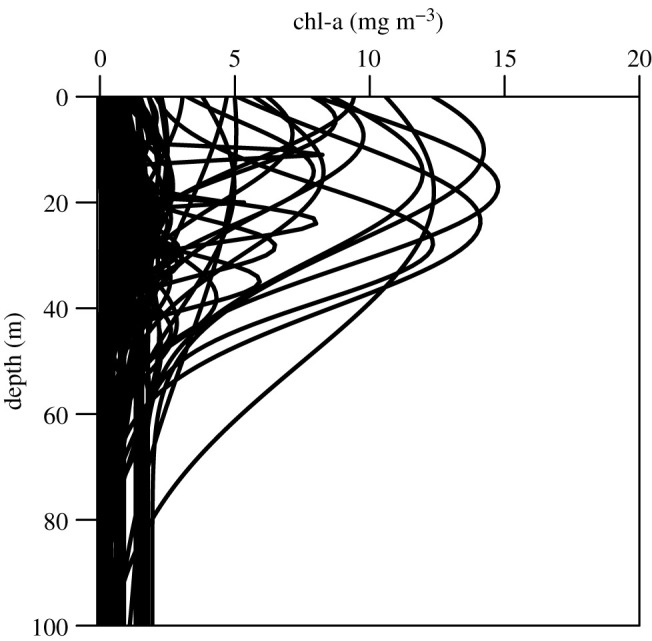
Shapes of chlorophyll profiles from the Labrador, Norwegian and Greenland Seas. Profiles were derived by fitting a four-parameter shifted Gaussian function to field observations [8].

**Figure 2.**
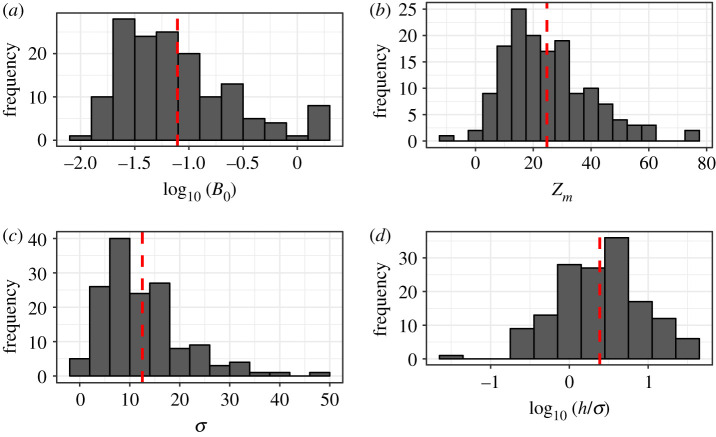
Histograms of chlorophyll profile parameters for the combined profile dataset. (*a*) *B*_0_ represents the background chlorophyll concentration, (*b*) *z_m_* is the depth of the peak chlorophyll concentration, (*c*) *σ* is the width of the Gaussian and (*d*) *h*/*σ* indicates the steepness of the slope of the peak. Vertical dashed lines indicate mean parameter values (*B*_0_ = 0.19, *z_m_* = 24.7, *σ* = 12.42, *h*/*σ* = 4.91). (Online version in colour.)

**Figure 3.**
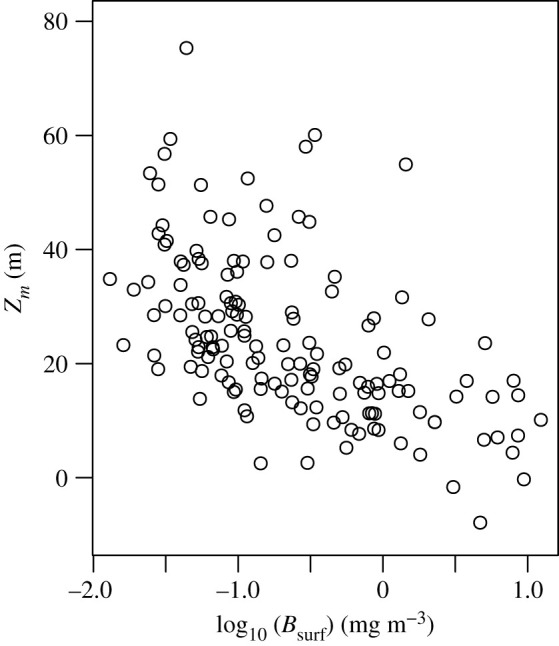
Relationship between surface chlorophyll concentration (*B*_surf_) and the depth of the subsurface chlorophyll maximum (*z_m_*) (*R*^2^ = 0.14, *p* < 0.001).

The shapes of the chlorophyll profiles varied in terms of the depth and width of the peak as well as the magnitude of the maximum chlorophyll concentration (figure 1). The average depth of the SCM (*z_m_*) was 24.7 m and ranged from −7.88 to 75.33 m (figure 2*b*), which is consistent with other pan-Arctic datasets [3–5] and reflects the large fraction of observations made during the post-bloom stratified period. Note that in a few rare cases, in order to achieve the best empirical fit to the chlorophyll measurements *z_m_* was situated slightly above the sea surface resulting in a small negative value. The background chlorophyll concentration *B*_0_ averaged 0.19 mg chl m^−3^ (figure 2*a*) and ranged from 0.01 to 1.94 mg chl m^−3^. The integral of the chlorophyll concentration under the Gaussian curve divided by the width of the chlorophyll peak (h/σ) also varied, with values ranging from 0.03 to 35.4 mg chl m^−3^ (figure 2*d*). There was a weak negative relationship between the surface chlorophyll concentration (*B*_surf_) and *z_m_* (figure 3) (*R*^2^ = 0.14, *p* < 0.001), which underscores the difficulty of using surface biomass as a predictor of the vertical structure of chlorophyll concentration in Arctic marine ecosystems [3,4].

**Figure 4.**
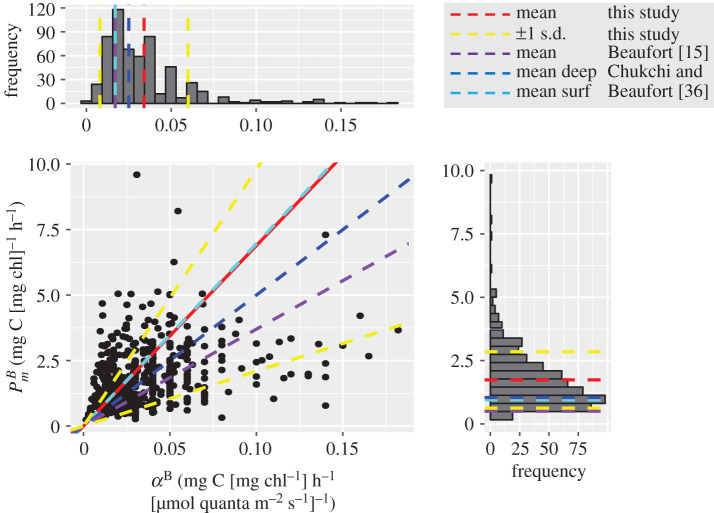
Correlation between the two PE parameters, the assimilation number ($P_{m}^{B}$) and the initial slope (*α*^*B*^), and their corresponding frequency distributions for the Arctic dataset used in this study. Red lines indicate mean parameter values for this study, blue and cyan dashed lines represent deep and surface assemblages in the Chukchi and Beaufort Seas [36], and purple dashed all samples from the Beaufort Sea [15]. Yellow lines represent ±1 s.d. from the mean value for this study.

The PE parameters also showed significant variability in this sector of the Arctic Ocean (figure 4). The average value of the light-saturated photosynthetic rate $P_{m}^{B}$ was 1.73 mg C (mg chl)^−1^ h^−1^ and ranged from 0.24 to 9.6 mg C (mg chl)^−1^ h^−1^. The mean value for $P_{m}^{B}$ is slightly higher than those reported in datasets from the Chukchi [0.95 mg C (mg chl)^−1^ h^−1^ for surface assemblages and 1.04 mg C (mg chl)^−1^ h^−1^ for SCM] and Beaufort [0.5 mg C (mg chl)^−1^ h^−1^ for all sampling depths] Seas [15,36] but this is likely due in part to the large spatial and temporal extent of this dataset. The initial slope *α*^*B*^ had a mean value of 0.034 mg C (mg chl)^−1 ^h^−1^ ${\left(\text{μ}{\text{mol quanta m}}^{-2}{\text{s}}^{-1}\right)}^{-1}$ and ranged from 0.002 to 0.182 (mg chl)^−1^ h^−1^ ${\left(\text{μ}\text{mol quanta}{\;}^{-2}{\text{s}}^{-1}\right)}^{-1}$. The average *α*^*B*^ value was higher than those reported for the Chukchi [0.017 mg C (mg chl)^−1^ h^−1^ for surface assemblages and 0.025 mg C (mg chl)^−1^ h^−1^ for SCM] and Beaufort [0.017 mg C (mg chl)^−1^ h^−1^] Seas [15,36]. The light adaptation parameter ($E_{k}=P_{m}^{B}/\alpha^{B})$ is a useful measure of the photoacclimatory status of the phytoplankton community. *E_k_* averaged 68 $\text{μ}{\text{mol quanta m}}^{-2}\;{\text{s}}^{-1}$ and ranged from 4 to 430 $\text{μ}{\text{mol quanta m}}^{-2}\;{\text{s}}^{-1}$. The average and range of values are consistent with those reported by Palmer and co-workers in the Chukchi and Beaufort Seas [36] and the mean value is nearly twice as high as that reported by Huot and co-workers for the Beaufort Sea [15], which may in part reflect the differences in the number of experiments conducted on surface and deep assemblages across the various datasets.

**Figure 5.**
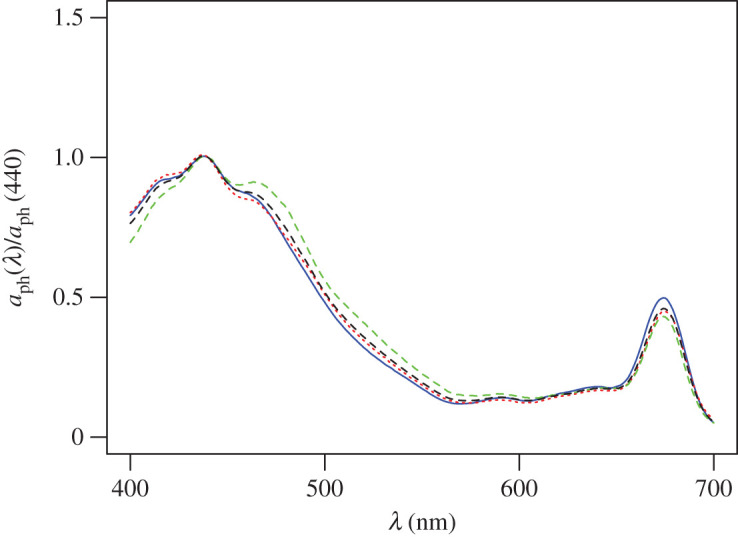
Mean *in vivo* light absorption spectra normalized to 440 nm (*a*_ph_(440)) for natural phytoplankton assemblages from the Barents (blue solid line), Labrador (red dotted line) and Greenland/Norwegian Seas (green dashed line). The black dashed line shows the average shape for the Atlantic Arctic region used in the primary production model to represent both phytoplankton light absorption and the spectral dependence of light-limited photosynthesis.

The average shape of the *in vivo* absorption spectrum of phytoplankton was remarkably similar across the three datasets (Greenland/Norwegian, Barents and Labrador Seas) (figure 5). The Greenland/Norwegian Sea dataset had a small shoulder in the blue-green region which was likely caused by differences in the taxonomic composition in the regional assemblages. Given the similarity of the spectral shape of $a_{p}(\lambda )$ over the study region, we used a single mean spectrum to represent the spectral dependence of both the absorption of light by phytoplankton and the light-limited photosynthesis [*α*^*B*^(*λ*)] in the primary production model.

The factors governing the formation of SCM layers within the global ocean have been explored in a recent review by Cullen [11]. In the Arctic Ocean, their prevalence during the post-bloom stratified period is driven by a combination of a freshening of surface waters caused by sea-ice melt and seasonal thermal stratification. This sets up ideal conditions for examining the impact of the vertical structure of chlorophyll on marine primary production over a broad range of stratification scenarios [37] with some water columns being characterized by shallow and in some cases intense and productive surface and subsurface chlorophyll layers, while others are more well-mixed resulting in phytoplankton biomass and productivity being more uniformly distributed. We used *in situ* bio-optical data and a vertically and spectrally resolved production model to identify the key parameters that are critical for accurate assessments of SCM productivity. For our initial model set-up, average PE parameters were assigned based on measurements made on natural Atlantic Arctic assemblages: $P_{m}^{B}$ and *α*^*B*^ were assigned values of 1.74 [mg C (mg chl)^−1^ h^−1^] and 0.034 [mg C (mg chl)^−1^ h^−1^ (µmol quanta m^−2^ s^−1^)^−1^], respectively. We first examined the difference between computed rates of daily primary production (DPP) when we held the surface concentration of chlorophyll constant with depth (uniform profile) and when we allowed chlorophyll to vary with depth using profile parameters obtained from fitting the nonlinear Gaussian function to Arctic field data (non-uniform profile) (figures 1 and 2).

**Figure 6.**
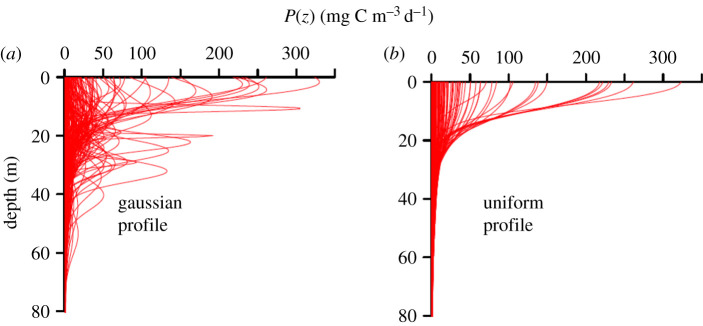
Profiles of estimates of daily primary production using (*a*) chlorophyll profiles shown in figure 1 and (*b*) uniform chlorophyll profile where the surface concentration is used for the entire water column. (Online version in colour.)

Figure 6 shows the estimated DPP profiles for both the uniform and non-uniform profile treatments. Note that the estimates of DPP at the sea surface are identical for both model runs but diverge with depth: water columns characterized by strong SCMs showed pronounced subsurface primary production peaks whereas the DPP profiles generated by holding the surface chlorophyll constant tend to decrease rapidly and exhibit the characteristic ‘half-wineglass' shape. Generally, production profiles characterized by a strong subsurface peak were associated with very thin chlorophyll layers located below 10 m.

**Figure 7.**
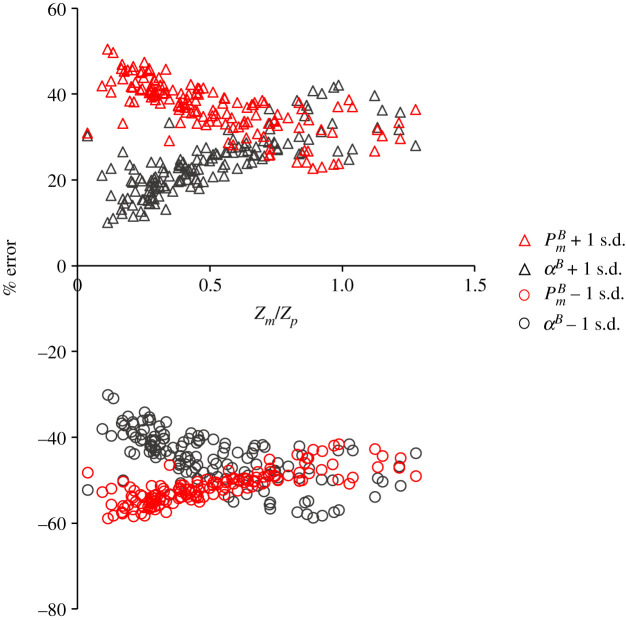
Results of a sensitivity analysis where the photosynthetic parameters $P_{m}^{B}$ and *α*^*B*^ were adjusted by ±1 s.d.. Per cent difference between model estimates of integrated production using mean and adjusted parameters are plotted against the depth of the subsurface chlorophyll peak divided by the depth of the euphotic zone (*z_m_/z_p_*). (Online version in colour.)

To examine how uncertainty in our assignment of the PE parameters may impact estimates of primary production for regions characterized by a diverse range of profile shapes we adjusted the PE parameters by ±1 s.d., which accounts for a large fraction of the seasonal and geographical variability in our Atlantic Arctic dataset, and also encompassed the average values obtained from other geographical sectors of the Arctic Ocean (figure 4). Errors in the estimation of water-column primary production were strongly related to the ratio of *z_m_* to *z_p_* (figure 7), which is an index of the vertical position of the chlorophyll peak relative to the photic depth. An overestimation ($P_{m}^{B}$ + 1 s.d.) of the light-saturation parameter $P_{m}^{B}\;\text{caused}$ a 23–50% overestimation of integrated primary production and there was a strong negative relationship between the per cent error and *z_m_/z_p_*. When most of the pigment biomass (*B*) is situated in the upper part of the photic zone (i.e. low *z_m_/z_p_*), the resulting biomass-normalized production rate (*P*^*B*^) as defined by the curvilinear PE response curve (equation (2.5)) approaches the plateau $P_{m}^{B}$ and errors will be maximal. However, in cases when most of the chlorophyll biomass is situated close to the bottom of the photic zone where the conditions are light-limiting, *P*^*B*^ will be governed by the quasi-linear region of the curve leading to estimates of *P*^*B*^ that are much lower than $P_{m}^{B}$ and consequently the negative error in primary production will be correspondingly lower. The degree to which the maximum photosynthetic rate is underestimated ($P_{m}^{B}$ − 1 s.d.) is also strongly governed by *z_m_/z_p_*, and again errors are highest in the upper portion of the water column where light is saturating and thus $P_{m}^{B}$ is governing the rate of *P*^*B*^. The inverse is true for errors in the initial slope, *α*^*B*^: when the initial slope is overestimated (*α*^*B*^ + 1 s.d.) or underestimated (*α*^*B*^ − 1 s.d.) we find the largest errors occur when most of the biomass is contained within the light-limited region of the euphotic zone (high *z_m_/z_p_*).

Clearly our choice of photosynthetic parameters directly impacts our estimates of marine primary production. Yet in several remote sensing studies of the Arctic Ocean a single $P_{m}^{B}$ value of 2 mg C (mg chl)^−1^ h^−1^ is often used [38] and is close to the mean value of 1.73 mg C (mg chl)^−1^ h^−1^ obtained in this study. Part of the difficulty in trying to capture the seasonal variability in the photophysiological properties of Arctic assemblages in primary production models is that there is no consensus on how to assign the PE parameters in an operational manner. Temperature [20,38], nutrient availability [36] and diatom dominance [15] have all been identified as potential predictor variables of $P_{m}^{B}$ in regional studies, but it would appear that no single relationship is robust across all geographical sectors. Moreover, from the point of view of implementing a method of parameter assignment for remote sensing applications, of the variables listed above, only sea-surface temperature can be retrieved directly from space. As the gaps in our knowledge of the biotic and abiotic factors governing variation in the PE parameters are slowly being filled by conventional ^14^C productivity experiments conducted at sea, there is also the potential of acquiring information on the photosynthetic characteristics of marine phytoplankton in a non-intrusive manner and over wider spatio-temporal scales using single turnover active chlorophyll fluorometry [39], although the conversion of electron transport rates into primary productivity estimates remains a challenge [23,40,41].

**Figure 8.**
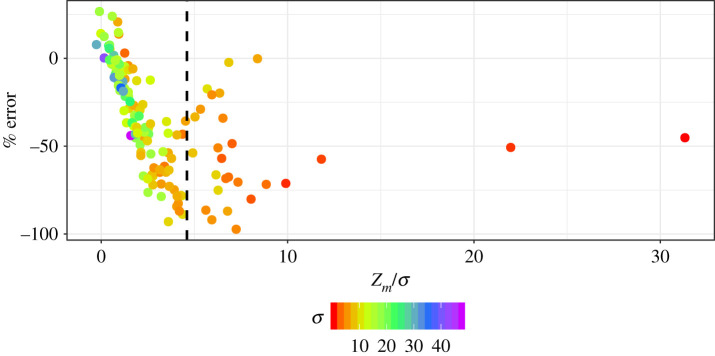
The per cent difference between model estimates of integrated primary production using a non-uniform and a uniform (surface chlorophyll concentration is held constant with depth) chlorophyll profile plotted against the depth of the subsurface chlorophyll peak divided by the peak width (*z_m_*/*σ*). Points are coloured according to their corresponding *σ* value. (Online version in colour.)

It has been argued that satellite algorithms that use surface chlorophyll concentrations may significantly underestimate water-column primary production in the Arctic Ocean [38]. Through examination of the equations governing primary production models, it is clear that the impact of SCMs on our estimates of integrated primary production is ultimately determined by whether the SCM is contained within the light-saturated or light-limited fraction of the photic zone. This means that knowing the shape of the chlorophyll profile is crucial. To represent the large range of profile shapes in our dataset, we decided to use the ratio of two of the profile parameters, the depth of the chlorophyll peak and its corresponding width (*z_m_*/*σ*), as our shape index. Note that in order to achieve the best nonlinear fit, *z_m_* may be assigned a value above the sea surface and as a result some *z_m_*/*σ* values were slightly negative. As an index of the relative contribution of SCMs to integrated primary production, we computed the per cent difference between model estimates when we include the profile parameters and when the chlorophyll was assumed to be uniformly distributed with depth. When we plot the relative change in primary production against *z_m_*/*σ* (figure 8), we see a very strong negative correlation for stations where *z_m_*/*σ* < 4.6 (*R*^2^ = 0.77, *p* < 0.01, figure 8) and a large scatter of points at higher values of *z_m_*/*σ*. To indicate which of the two profile parameters is responsible for the anomalously high values of *z_m_*/*σ*, we also coloured each point according to its corresponding *σ* value.

**Figure 9.**
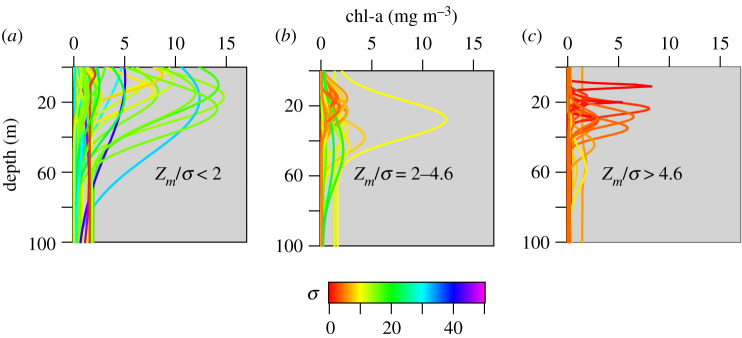
Chlorophyll profiles shown in figure 1 grouped according to their corresponding *z_m_*/*σ* value: (*a*) <2, (*b*) 2–4.6 and (*c*) >4.6. Lines are coloured according to the *σ* value for each chlorophyll profile. (Online version in colour.)

Figure 8 clearly shows the impact the vertical structure of chlorophyll has on our estimates of integrated primary production: when *z_m_*/*σ* is low, the peak is confined in the upper part of the water column where light levels are sufficient to support high rates of productivity. In cases where the centre of the Gaussian peak is slightly above or very close to the sea surface, the integrated biomass will be overestimated if a uniform profile is applied, since the estimated productivity profile using the non-uniform chlorophyll profile will attenuate more rapidly with depth. Note also that, in most cases, the peaks were much broader than deeper profiles with higher *z_m_*/*σ* values, as indicated by the high *σ* values. Although there tends to be a general pattern of SCM peaks closer to the surface to be broader, which will more closely resemble a uniform biomass profile in the upper portion of the water column where primary production rates will be highest, there is still significant variability in profile shape, which is illustrated in figure 9*a*. Intermediate *z_m_*/*σ* values (between 2 and 4.6) result from the peak being situated deeper in the water-column (higher *z_m_*) (figure 9*b*), but as the peak width is narrower, a large fraction of the chlorophyll biomass is still contained within the euphotic zone. The presence of deeper and more prominent peaks leads to the SCM accounting for a significant fraction of the integrated primary production as indicated by the large difference between the estimates using uniform and non-uniform profiles and relative errors reaching a maximum of 98%.

Another interesting feature of figure 8 is that at very high *z_m_*/*σ* values the strong relationship between our index of SCM contribution to integrated primary production and profile shape breaks down. There are two scenarios that lead to this result. First is the situation where a thin chlorophyll layer is present (figure 9*c*), which leads to anomalously high *z_m_*/*σ* values. Even though these profiles exhibited some of the highest concentrations in subsurface chlorophyll and highest rates of primary production per unit volume, when integrated over the water column, their impact on integrated rates of primary production was not as high as subsurface peaks that were broader but less intense. The other situation was profiles which had a very small bump superimposed on a uniform background concentration (figure 9*c*). Although both of these cases are relatively rare in our dataset, it should be noted that the presence of thin subsurface chlorophyll layers can be a common occurrence in temperate shelf seas [42] and the stratification conditions that lead to their formation may not be well represented in the hydrographic regions represented in this study. Nevertheless, the relationship between profile shape and the SCM contribution to water-column primary production serves as a useful means of classifying SCMs in terms of their significance to Arctic Ocean productivity.

**Figure 10.**
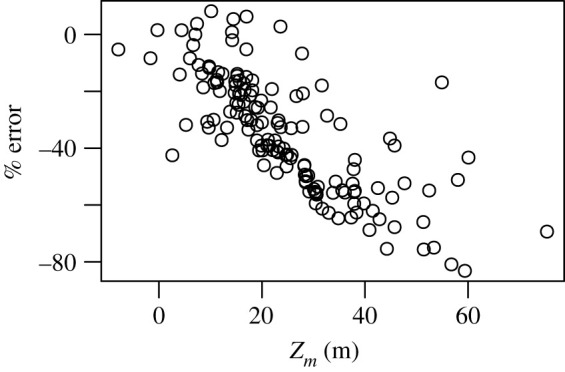
Per cent error in the estimation of integrated primary production when CDOM absorption coefficient at 440 nm is increased to a uniform background value of 0.07 nm^−1^ (*R*^2^ = 0.58, *p* < 0.001).

We also examined how changes in light availability caused by cloud cover and absorption by CDOM impact estimates of daily water-column primary production when a non-uniform profile is applied. In the case of errors caused by cloud cover, we found a maximum error of up to 16% when the clear-sky model is used under conditions of 100% cloud cover using our standard model run. Similarly, when we adjusted the CDOM absorption to a constant background value of 0.07 m^−1^, which can be considered an extreme case when surface waters are heavily influenced by terrigenous sources, estimates of integrated primary production decreased by as much as 83% and this was a strong function of the depth of the chlorophyll peak (*R*^2^ = 0.58, *p* < 0.001, figure 10). Thus, in order to assess the role SCM primary production plays in Arctic marine environments relies on an accurate description of the underwater light field, which in some optically complex sectors of the Arctic Ocean is difficult to model without detailed information of the absorption by non-algal particulate and dissolved substances. When considering the estimation of primary production using ocean-colour remote sensing, the presence of CDOM in the surface waters of the Arctic not only impacts our estimate of light attenuation but also our retrieval of surface chlorophyll concentration [2]. Although the overestimation of chlorophyll by satellite due to the presence of CDOM may counterbalance the underestimation of integrated primary production by ignoring the presence of SCMs [2], these two potential sources of error make the overall interpretation of large-scale temporal trends in primary production challenging, especially as both CDOM supply and SCM prevalence may change in a future Arctic Ocean. Yet, in the case of computations of integrated primary production using satellite remote sensing reflectance over annual and basin scales, adopting a simple approach to describe vertical changes in chlorophyll biomass may lead to smaller errors than a more elaborate one, given our current limitations to accurately predict either the vertical structure of pigment biomass or the relative contribution of CDOM to the ocean-colour signal.

Given that SCMs are considered to be ubiquitous across the Arctic ecosystem over a large fraction of the growth season, there is a need to apply realistic profile shapes in order to assess their role in marine primary production. To address the uneven distribution and sparsity of chlorophyll measurements in the Arctic Ocean, most assessments of the contribution of SCMs to Arctic Ocean productivity take the approach of averaging profile data both spatially and temporally. Our model results suggest that such spatial and temporal averaging will likely lead to an underestimation of the importance of SCMs by altering the depth-dependent variability through flattening and broadening the peaks. Fortunately, our ability to observe SCMs in remote Arctic marine systems will dramatically improve in the coming years. Although SCMs are invisible to ocean-colour sensors, there is the exciting prospect that new Lidar sensors will be able to detect these features from space [43]. Meanwhile, the increased use of ice-tethered profilers [44], Bio-ARGO floats [45], animal-borne instruments [46] and autonomous underwater vehicles [47,48] will allow us to monitor the seasonal dynamics of SCM vertical structure at an unprecedented spatial resolution. Despite the difficulty in obtaining a precise measure of chlorophyll concentration from fluorescence sensors on such monitoring platforms [11], especially in the Arctic where *in situ* validation is in many cases impossible, this study demonstrates that if we are provided with qualitative information on the relative shape of chlorophyll profiles from these rich data streams, we can begin to make significant progress in evaluating the role of SCMs in the Arctic Ocean carbon cycle.

Determining rates of primary productivity within SCMs is not only critical to improve our estimates of water-column productivity but also is essential for developing a better understanding of the capacity of Arctic ecosystems to sustain secondary and higher trophic levels, which in turn is ultimately determined by the rate of the supply of new nutrients from outside the photic zone and the rate of organic matter being removed through sedimentation [49]. The widespread prevalence of SCMs is the direct result of the supply of new nutrients into the base of the photic zone [5] and this intimate link between new nutrients and SCM productivity is reflected by the depth of the SCM coinciding with that of the nutricline. It has been argued that inferring rates of export production from those of new production is problematic in the Arctic Ocean, since the downward flux of organic material out of the euphotic zone is a function of both gravitational and injection pumps that regulate the vertical transport of marine particles [50] and that the physically mediated bottom-up processes regulating rates of new production may operate over different temporal and spatial scales from the top-down processes that govern the transport of organic matter out of the photic zone. Yet the evidence of elevated export rates in the vicinity of the SCM under stratified conditions [51] and a community of grazers situated towards the base of the photic zone [37] supports the view that SCMs are important in pelagic-benthic coupling during the post-bloom vegetative season.

SCMs have also been shown to be important ecological niches that host diverse phytoplankton assemblages. Within these layers, the growth of larger cells (e.g. diatoms) can be sustained by the subsurface nutrient reservoir while at the same time these algae are able to harvest sufficient light despite being prone to high pigment packaging [4,22]. Even within the smallest size fraction of the phytoplankton community, changes in phylogenetic composition within SCMs can be observed [52]. A study using high-throughput sequencing of microbial communities has shown that strongly stratified water columns with a sharp SCM tend to have a high proportion of *Micromonas* (prasinophyceae) reads. As one of the smallest photosynthetic eukaryotic organisms in the Arctic Ocean, it has been suggested that *Micromonas* may be used as a sentinel of a changing Arctic Ocean [53,54]. Thus, understanding the diversity and productivity of SCMs will be essential to understanding the ecological consequences of a rapidly changing Arctic Ocean that is evolving towards a more stratified and nutrient-limited system.

## 4. Summary and conclusion

Here, we show that the importance of the relative contribution of SCMs to water-column productivity is strongly controlled by the shape of the chlorophyll profile and that a failure to account for the presence of SCMs can lead to a significant underestimate in water-column primary production, with relative errors approaching 100%. This highlights the importance of capturing detailed information on the vertical structure of SCMs and demonstrates that spatio-temporal averaging of chlorophyll profiles could lead to an underestimation of the contribution of SCMs to Arctic primary production. A variety of autonomous platforms that are able to record the vertical distribution of chlorophyll fluorescence at a high depth resolution will provide an unprecedented view of the geographical extent of SCMs and their structural diversity and provide invaluable insight into the environmental factors that control their properties and distribution.

Previous modelling studies have shown that errors introduced by ignoring the presence of SCMs when computing annual rates of primary production over ocean basins or provinces are modest (<10%), and that the impact of the vertical structure of chlorophyll on estimates of integrated rates can be considered of secondary importance [2,5] when compared with errors in the retrieval of surface chlorophyll, which has been shown to be a problem in optically complex sectors of the Arctic Ocean [2]. Yet future changes in the physical factors governing nutrient supply and light availability may cause the relative importance of SCMs to increase [55]. Moreover, as SCMs are sustained by the vertical supply of nutrients, they are also important contributors to new production [38] for a large portion of the growth season and for this reason the estimation of primary production within SCMs may be more important than gross primary production for the purposes of climate studies [5,38]. SCMs also coincide with the region of the water column where the highest rates of particle flux are often observed [51]. Given our aim is to develop insight into the response of the Arctic pelagic system to climate change, systematic errors introduced by models that do not include SCMs could hinder our ability to accurately detect long-term trends in Arctic Ocean productivity and assess its ecological and biogeochemical consequences.

## Supplementary Material

Table S1

## Supplementary Material

Table S2

## Supplementary Material

Table S3

## Data accessibility

The datasets supporting this article have been uploaded as part of the electronic supplementary material. The PE parameters included in the electronic supplementary material section are a subset of two datasets: a global dataset [14], which is publically available (https://doi.org/10.1594/PANGAEA.874087) and one on the Labrador Sea [29], which is publically available at https://doi.org/10.1594/PANGAEA.871872. Model data are available by contacting H.A.B.

## References

[1] Martin J et al 2010 Prevalence, structure and properties of subsurface chlorophyll maxima in Canadian Arctic waters. Mar. Ecol. Prog. Ser. 412, 69–84. (10.3354/meps08666)

[2] Arrigo KR, Matrai PA, Van Dijken GL. 2011 Primary productivity in the Arctic Ocean: impacts of complex optical properties and subsurface chlorophyll maxima on large-scale estimates. J. Geophys. Res. Ocean. 116, C11022 (10.1029/2011JC007273)

[3] Hill VJ, Matrai PA, Olson E, Suttles S, Steele M, Codispoti LA, Zimmerman RC 2013 Synthesis of integrated primary production in the Arctic Ocean: II. In situ and remotely sensed estimates. Prog. Oceanogr. 110, 107–125. (10.1016/j.pocean.2012.11.005)

[4] Ardyna M, Babin M, Gosselin M, Devred E, Bélanger S, Matsuoka A, Tremblay JE 2013 Parameterization of vertical chlorophyll a in the Arctic Ocean: impact of the subsurface chlorophyll maximum on regional, seasonal, and annual primary production estimates. Biogeosciences 10, 4383–4404. (10.5194/bg-10-4383-2013)

[5] Sathyendranath S, Longhurst A, Caverhill CM, Platt T 1995 Regionally and seasonally differentiated primary production in the North Atlantic. Deep. Res. Part I 42, 1773–1802. (10.1016/0967-0637(95)00059-F)

[6] Cherkasheva A, Nöthig E-M, Bauerfeind E, Melsheimer C, Bracher A 2013 From the chlorophyll a in the surface layer to its vertical profile: a Greenland Sea relationship for satellite applications. Ocean Sci. 9, 431–445. (10.5194/os-9-431-2013)

[7] Matrai PA, Olson E, Suttles S, Hill V, Codispoti LA, Light B, Steele M 2013 Synthesis of primary production in the Arctic Ocean: I. Surface waters, 1954–2007. Prog. Oceanogr. 110, 93–106. (10.1016/j.pocean.2012.11.004)

[8] Platt T, Sathyendranath S, Caverhill CM, Lewis MR 1988 Ocean primary production and available light: further algorithms for remote sensing. Deep Sea Res. Part A Oceanogr. Res. Pap. 35, 855–879. (10.1016/0198-0149(88)90064-7)

[9] Brown ZW, Lowry KE, Palmer MA, van Dijken GL, Mills MM, Pickart RS, Arrigo KR. 2015 Characterizing the subsurface chlorophyll a maximum in the Chukchi Sea and Canada Basin. Deep. Res. Part II Top. Stud. Oceanogr. 118, 88–104. (10.1016/j.dsr2.2015.02.010)

[10] Herbland A, Voituriez B 1979 Hydrological structure analysis for estimating the primary production in the tropical Atlantic Ocean. J. Mar. Res. 37, 87–101.

[11] Cullen JJ 2015 Subsurface chlorophyll maximum layers: enduring enigma or mystery solved? Ann. Rev. Mar. Sci. 7, 207–239. (10.1146/annurev-marine-010213-135111) 25251268

[12] Jassby AD, Platt T 1976 Mathematical formulation of the relationship between photosynthesis and light for phytoplankton. Limnol. Oceanogr. 21, 540–547. (10.4319/lo.1976.21.4.0540)

[13] Geider RJ, MacIntyre HL, Kana TM 1997 Dynamic model of phytoplankton growth and acclimation: responses of the balanced growth rate and the chlorophyll a: carbon ratio to light, nutrient-limitation and temperature. Mar. Ecol. Prog. Ser. 148, 187–200. (10.3354/meps148187)

[14] Bouman HA et al 2018 Photosynthesis-irradiance parameters of marine phytoplankton: synthesis of a global data set Earth Syst. Sci. Data 10, 251–266. (10.5194/essd-10-251-2018)

[15] Huot Y, Babin M, Bruyant F 2013 Photosynthetic parameters in the Beaufort Sea in relation to the phytoplankton community structure. Biogeosciences 10, 3445–3454. (10.5194/bg-10-3445-2013)

[16] Li W, Smith J, Platt T 1984 Temperature response of photosynthetic capacity and carboxylase activity in Arctic marine phytoplankton. Mar. Ecol. Prog. Ser. 17, 237–243. (10.3354/meps017237)

[17] Longhurst A, Sathyendranath S, Platt T, Caverhill C 1995 An estimate of global primary production in the ocean from satellite radiometer data. J. Plankton Res. 17, 1245–1271. (10.1093/plankt/17.6.1245)

[18] Lee YJ et al 2015 An assessment of phytoplankton primary productivity in the Arctic Ocean from satellite ocean color/in situ chlorophyll-a based models. J. Geophys. Res. Ocean. 120, 6508–6541. (10.1002/2015JC011018) PMC5014238 27668139

[19] Gallegos CL, Platt T, Harrison WG, Irwin B 1983 Photosynthetic parameters of Arctic marine phytoplankton: vertical variations and time scales of adaptation. Limnol. Oceanogr. 28, 698–708. (10.4319/lo.1983.28.4.0698)

[20] Harrison WG, Platt T 1986 Photosynthesis-irradiance relationships in polar and temperate phytoplankton populations. Polar Biol. 5, 153–164. (10.1007/BF00441695)

[21] Cota GF, Smith WO, Mitchell BG 1994 Photosynthesis of Phaeocystis in the Greenland Sea. Limnol. Oceanogr. 39, 948–953. (10.4319/lo.1994.39.4.0948)

[22] Palmer MA et al 2011 Spatial and temporal variation of photosynthetic parameters in natural phytoplankton assemblages in the Beaufort Sea, Canadian Arctic. Polar Biol. 34, 1915–1928. (10.1007/s00300-011-1050-x)

[23] Schuback N, Hoppe CJM, Tremblay JÉ, Maldonado MT, Tortell PD 2017 Primary productivity and the coupling of photosynthetic electron transport and carbon fixation in the Arctic Ocean. Limnol. Oceanogr. 62, 898–921. (10.1002/lno.10475)

[24] Kishino M, Takahashi M, Okami N, Ichimura S 1985 Estimation of the spectral absorption coefficients of phytoplankton in the sea. Bull. Mar. Sci. 37, 634–642.

[25] Hoepffner N, Sathyendranath S 1992 Bio-optical characteristics of coastal waters: absorption spectra of phytoplankton and pigment distribution in the western North Atlantic. Limnol. Oceanogr. 37, 1660–1679. (10.4319/lo.1992.37.8.1660)

[26] Cota GF, Harrison WG, Platt T, Sathyendranath S, Stuart V 2003 Bio-optical properties of the Labrador Sea. J. Geophys. Res. Atmos. 108, 21 (10.1029/2000jc000597)

[27] Platt T, Gallegos CL, Harrison WG 1980 Photoinhibition of photosynthesis in natural assemblages of marine phytoplankton. J. Mar. Res. 38, 687–701.

[28] Kyewalyanga MN, Platt T, Sathyendranath S 1997 Estimation of the photosynthetic action spectrum: implication for primary production models. Mar. Ecol. Prog. Ser. 146, 207–223. (10.3354/meps146207)

[29] Fragoso GM, Poulton AJ, Yashayaev IM, Head EJH, Purdie DA 2017 Spring phytoplankton communities of the Labrador Sea (2005–2014): pigment signatures, photophysiology and elemental ratios Biogeosciences 14, 1235–1259. (10.5194/bg-14-1235-2017)

[30] Kyewalyanga M, Platt T, Sathyendranath S 1992 Ocean primary production calculated by spectral and broad-band models. Mar. Ecol. Prog. Ser. 85, 171–185. (10.3354/meps085171)

[31] Bouman HA, Platt T, Sathyendranath S, Irwin BD, Wernand MR, Kraay GW 2000 Bio-optical properties of the subtropical North Atlantic. II. Relevance to models of primary production. Mar. Ecol. Prog. Ser. 200, 19–34. (10.3354/meps200019)

[32] Sathyendranath S, Platt T 1989 Computation of aquatic primary production: extended formalism to include effect of angular and spectral distribution of light. Limnol. Oceanogr. 34, 188–198. (10.4319/lo.1989.34.1.0188)

[33] Smith EL 1936 Photosynthesis in relation to light and carbon dioxide. Proc. Natl Acad. Sci. USA 22, 504–511. (10.1073/pnas.22.8.504) 16577734 PMC1079215

[34] Bird RE 1984 A simple, solar spectral model for direct-normal and diffuse horizontal irradiance. Sol. Energy 32, 461–471. (10.1016/0038-092X(84)90260-3)

[35] Platt T, Sathyendranath S, Ravindran P 1990 Primary production by phytoplankton: analytic solutions for daily rates per unit area of water surface. Proc. R. Soc. B 241, 101–111. (10.1098/rspb.1990.0072)

[36] Palmer MA, van Dijken GL, Greg Mitchell B, Seegers BJ, Lowry KE, Mills MM, Arrigo KR. 2013 Light and nutrient control of photosynthesis in natural phytoplankton populations from the Chukchi and Beaufort seas, Arctic Ocean. Limnol. Oceanogr. 58, 2185–2205. (10.4319/lo.2013.58.6.2185)

[37] Carmack E, Wassmann P 2006 Food webs and physical-biological coupling on pan-Arctic shelves: unifying concepts and comprehensive perspectives. Prog. Oceanogr. 71, 446–477. (10.1016/j.pocean.2006.10.004)

[38] Martin J, Tremblay JÉ, Price NM 2012 Nutritive and photosynthetic ecology of subsurface chlorophyll maxima in Canadian Arctic waters. Biogeosciences 9, 5353–5371. (10.5194/bg-9-5353-2012)

[39] Boatman TG, Geider RJ, Oxborough K 2019 Improving the accuracy of single turnover active fluorometry (STAF) for the estimation of phytoplankton primary productivity (PhytoPP). Front. Mar. Sci. 6, 1–16. (10.3389/fmars.2019.00319)

[40] Lawrenz E et al 2013 Predicting the electron requirement for carbon fixation in seas and oceans. PLoS ONE 8, e0058137 (10.1371/journal.pone.0058137) PMC3596381 23516441

[41] Suggett DJ, MacIntyre HL, Kana TM, Geider RJ 2009 Comparing electron transport with gas exchange: parameterising exchange rates between alternative photosynthetic currencies for eukaryotic phytoplankton. Aquat. Microb. Ecol. 56, 147–162. (10.3354/ame01303)

[42] Barnett ML, Kemp AES, Hickman AE, Purdie DA 2019 Shelf sea subsurface chlorophyll maximum thin layers have a distinct phytoplankton community structure. Cont. Shelf Res. 174, 140–157. (10.1016/j.csr.2018.12.007)

[43] Neukermans G et al 2018 Harnessing remote sensing to address critical science questions on ocean–atmosphere interactions. Elementa 6, 331 (10.1525/elementa.331)

[44] Laney SR, Krishfield RA, Toole JM, Hammar TR, Ashjian CJ, Timmermans ML 2014 Assessing algal biomass and bio-optical distributions in perennially ice-covered polar ocean ecosystems. Polar Sci. 8, 73–85. (10.1016/j.polar.2013.12.003)

[45] Roemmich D et al 2019 On the future of Argo: a global, full-depth, multi-disciplinary array. Front. Mar. Sci. 6, 1–28. (10.3389/fmars.2019.00439)

[46] March D, Boehme L, Tintoré J, Vélez-Belchi PJ, Godley BJ 2020 Towards the integration of animal-borne instruments into global ocean observing systems. Glob. Chang. Biol. 26, 586–596. (10.1111/gcb.14902) 31675456 PMC7027834

[47] Perry MJ, Sackmann BS, Eriksen CC, Lee CM 2008 Seaglider observations of blooms and subsurface chlorophyll maxima off the Washington coast. Limnol. Oceanogr. 53, 2169–2179. (10.4319/lo.2008.53.5_part_2.2169)

[48] Porter M, Henley SF, Orkney A, Bouman HA, Hwang B, Dumont E, Venables EJ, Cottier F 2020 A polar surface eddy obscured by thermal stratification. Geophys. Res. Lett. 47, 1–19. (10.1029/2019GL086281)

[49] Platt T, Jauhari P, Sathyendranath S 1992 The importance and measurement of new production. In Primary Productivity and Biogeochemical Cycles in the Sea. Environmental Science Research (eds Falkowski PG, Woodhead AD, Vivirito K), vol. 43 Boston, MA: Springer.

[50] Boyd PW, Claustre H, Levy M, Siegel DA, Weber T 2019 Multi-faceted particle pumps drive carbon sequestration in the ocean. Nature 568, 327–335. (10.1038/s41586-019-1098-2) 30996317

[51] Wassmann P, Reigstad M 2011 Future Arctic Ocean seasonal ice zones and implications for pelagic-benthic coupling. Oceanography 24, 220–231. (10.5670/oceanog.2011.74)

[52] Monier A, Comte J, Babin M, Forest A, Matsuoka A, Lovejoy C 2015 Oceanographic structure drives the assembly processes of microbial eukaryotic communities. ISME J. 9, 990–1002. (10.1038/ismej.2014.197) 25325383 PMC4817713

[53] Li WKW, McLaughlin FA, Lovejoy C, Carmack EC 2009 Smallest algae thrive as the arctic ocean freshens. Science 326, 539 (10.1126/science.1179798) 19900890

[54] Demory D et al 2019 Picoeukaryotes of the Micromonas genus: sentinels of a warming ocean. ISME J. 13, 132–146. (10.1038/s41396-018-0248-0) 30116039 PMC6299001

[55] Popova EE, Yool A, Coward AC, Aksenov YK, Alderson SG, De Cuevas BA, Anderson TR. 2010 Control of primary production in the Arctic by nutrients and light: insights from a high resolution ocean general circulation model. Biogeosciences 7, 3569–3591. (10.5194/bg-7-3569-2010)

